# Efficient Near-Infrared Luminescence Based on Double Perovskite Cs_2_SnCl_6_

**DOI:** 10.3390/molecules28083593

**Published:** 2023-04-20

**Authors:** Xiaofei Qing, Chuanli Wu, Xiuxun Han

**Affiliations:** 1 Institute of Optoelectronic Materials and Devices, Faculty of Materials Metallurgy and Chemistry, Jiangxi University of Science and Technology, Ganzhou 341000, China; 2 National Rare Earth Function Materials Innovation Center, Ganzhou 341100, China; 3 Guorui Scientific Innovation Rare Earth Functional Materials (Ganzhou) Co., Ltd., Ganzhou 341000, China

**Keywords:** Cs_2_SnCl_6_ perovskite, Te^4+^/Er^3+^ co-doped, NIR emissions, energy transfer

## Abstract

Cs_2_SnCl_6_ double perovskite has attracted wide attention as a promising optoelectronic material because of its better stability and lower toxicity than its lead counterparts. However, pure Cs_2_SnCl_6_ demonstrates quite poor optical properties, which usually calls for active element doping to realize efficient luminescence. Herein, a facile co-precipitation method was used to synthesize Te^4+^ and Er^3+^-co-doped Cs_2_SnCl_6_ microcrystals. The prepared microcrystals were polyhedral, with a size distribution around 1–3 μm. Highly efficient NIR emissions at 1540 nm and 1562 nm due to Er^3+^ were achieved in doped Cs_2_SnCl_6_ compounds for the first time. Moreover, the visible luminescence lifetimes of Te^4+^/Er^3+^-co-doped Cs_2_SnCl_6_ decreased with the increase in the Er^3+^ concentration due to the increasing energy transfer efficiency. The strong and multi-wavelength NIR luminescence of Te^4+^/Er^3+^-co-doped Cs_2_SnCl_6_ originates from the 4f→4f transition of Er^3+^, which was sensitized by the spin-orbital allowed ^1^S_0_→^3^P_1_ transition of Te^4+^ through a self-trapped exciton (STE) state. The findings suggest that ns^2^-metal and lanthanide ion co-doping is a promising method to extend the emission range of Cs_2_SnCl_6_ materials to the NIR region.

## 1. Introduction

The Pb-halide perovskite photovoltaics has been seen in the rapid rise of power conversion efficiency (PCE) in the past several years, expectedly contributing to sustainable development via green energy strategies [1,2]. In addition, Pb-halide perovskites have attracted increasing attention due to their outstanding photoelectric properties, such as their tunable band gap and high photoluminescence quantum yield (PLQY) [3,4,5]. However, there are growing concerns about the lead toxicity to human health and the environment [6,7]. Therefore, various efforts have been made to replace Pb with non-toxic metals, such as tin (Sn), germanium (Ge), bismuth (Bi), and indium (In) [8,9,10,11]. Among those alternative elements, Sn is deemed as a perfect choice because it has the most similar electronic properties in the same group of the periodic table with lead. As expected, tin halide perovskites (CsSnX_3_) can also offer an outstanding optoelectronic performance including their narrow band gap, low exciton binding energy, and long carrier diffusion length [12,13,14]. Unfortunately, CsSnX_3_ perovskites suffer from rather poor stability under ambient conditions due to the easy oxidation of Sn^2+^ to Sn^4+^ [15,16].

In such a context, Sn^4+^-based perovskite variants (Cs_2_SnX_6_) are much more stable than CsSnX_3_ perovskites [14]. However, Cs_2_SnX_6_ exhibits significantly inferior optical properties in the visible and near-infrared (NIR) regions compared to Pb-halide perovskites [17,18]. It is reported that the 6s^2^ electrons of Pb^2+^ play a major role in avoiding the formation of deep trap defects, resulting in highly efficient optoelectronic processes [19,20]. Whereas, the ns^2^ electronic configuration of Sn^4+^ is lost in Cs_2_SnX_6_. Therefore, doping elements with ns^2^ electrons is a very effective method to improve their optoelectronic performance [21]. For example, Bi^3+^-, Sb^3+^-, and Te^4+^-doped Cs_2_SnX_6_ have produced intense blue, red, and yellow emissions, respectively [22,23,24]. In addition, white light emission was obtained from Cs_2_SnX_6_ that was co-doped with Bi^3+^ and Te^4+^ ions [25,26]. Up until now, Cs_2_SnX_6_ luminescence has almost covered the whole visible region through ion doping. However, to the best of our knowledge, there are few reports of achieving luminescence in the NIR region in Sn^4+^-based perovskites, especially for Cs_2_SnCl_6_ with a wide band gap. While NIR is of significant importance in many applications, including night vision, thermal imaging, bioimaging, and wellness monitoring [27]. Therefore, it is critically demanding to achieve efficient NIR-emitting perovskite derivatives.

To achieve NIR luminescence in Cs_2_SnCl_6_, lanthanide (Ln^3+^) ions with proper emissions such as Er^3+^, Yb^3+^, and Nd^3+^ are good dopants [28,29,30]. However, a very high excitation energy is required in those Ln^3+^-doped compounds due to the parity forbidden transitions within the 4f^N^ configurations of Ln^3+^ [31,32]. Fortunately, ns^2^ doping can introduce new light absorption channels and thus act as sensitizers for luminescent Ln^3+^, such as Er^3+^, Yb^3+^, and Nd^3+^. For example, intense and multi-wavelength NIR luminescence was obtained in Cs_2_ZrCl_6_ at a low excitation energy through Te^4+^ co-doping with Er^3+^, Nd^3+^, or Yb^3+^ ions [33].

Herein, we realized the NIR emission in Te^4+^- and Er^3+^-co-doped Cs_2_SnCl_6_ microcrystal following a simple co-precipitation method. Under the low energy excitation at 391 nm, Te^4+^/Er^3+^-co-doped Cs_2_SnCl_6_ displays an efficient NIR emission peak at ~1540 nm, in contrast to a negligible emission peak at this position in Er^3+^-singly doped Cs_2_SnCl_6_. The energy transfer processes from Te^4+^ to Er^3+^ f-electrons are proposed and discussed in detail based on the experimental findings.

## 2. Results and Discussion

### 2.1. Crystal Structure and Characterization

**Figure 1:**
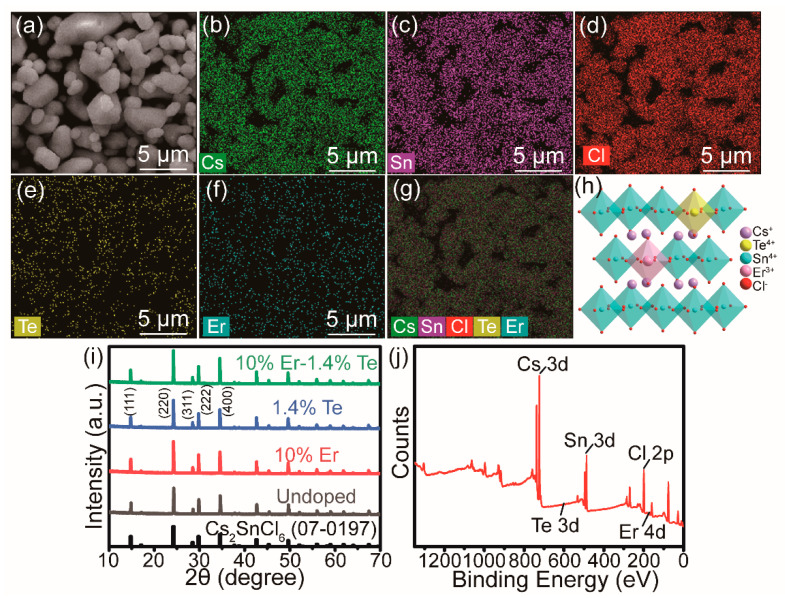
(**a**) SEM image, (**b**–**g**) EDS elemental mappings (Cs, Sn, Cl, Te, Er, and merge), and (**h**) crystal structure of cubic Te^4+^/Er^3+^-co-doped Cs_2_SnCl_6_. (**i**) XRD pattern of undoped, Er^3+^-singly doped, Te^4+^-singly doped, and Te^4+^/Er^3+^-co-doped Cs_2_SnCl_6_ microcrystals. The 2θ peaks located at 14.8°, 24.2°, 28.5°, 29.7°, and 34.5° correspond to the (111), (220), (311), (222), and (400) diffraction planes, respectively. (**j**) XPS survey spectrum of Te^4+^/Er^3+^-co-doped Cs_2_SnCl_6_ microcrystals.

The Te^4+^/Er^3+^-co-doped Cs_2_SnCl_6_ particles were synthesized through a facile co-precipitation method [33]. To be brief, the precursors SnCl_4_, TeO_2_, and ErCl_3_·6H_2_O were mixed with HCl and ethanol and dissolved. Thereafter, Cs_2_CO_3_ (dissolved in HCl) was added into the reaction mixture, and the perovskite MCs were immediately precipitated. More synthesis details are described in the Supporting Information (SI). As depicted in Figure 1a, the scanning electron microscopy (SEM) image showed that the size of the obtained Cs_2_SnCl_6_ crystals with a nominal molar concentration of 1.4% Te^4+^ and 10% Er^3+^ was mainly in the range of about 1–3 μm (Figure S1). The energy-dispersive spectroscopy (EDS) mapping in Figure 1b–g demonstrated that the constituent elements were uniformly distributed in the microcrystals, and the estimated Cs:(Sn+Te+Er):Cl composition ratio of microcrystals roughly agreed with the stoichiometric ratio of Cs_2_SnCl_6_, as given in Table S1. Moreover, the exact doping contents of Te^4+^ and Er^3+^ were determined to be 1.6% and 2.0% by inductively coupled plasma mass spectrometry (ICP-MS) (Table S2). It is noted that the Te^4+^ actual concentration was a little higher than its feeding concentration of 1.4%, which is mainly due to the high solubility of Te^4+^ in Cs_2_SnCl_6_ and the lower formation energy of Cs_2_TeCl_6_ than that of Cs_2_SnCl_6_ [34,35].

The XRD pattern in Figure 1h,i confirmed the cubic perovskite-type structure of Cs_2_SnCl_6_ with space group *Fm-3m* (PDF no. 07-0197), and no impurity phases were detected. In addition, the Rietveld analysis indicates that the diffraction peaks shifted to lower angles after doping (Figure S2) due to the lattice expansion (Table S3), as Sn^4+^ (r = 0.69 Å, CN = 6) was substituted by larger Te^4+^ (r = 0.97 Å, CN = 6) and Er^3+^ (r = 0.89 Å, CN = 6). These results indicated that Te^4+^, Er^3+^, and Te^4+^/Er^3+^ were successfully doped into the Cs_2_SnCl_6_ crystal lattice. X-ray photoelectron spectroscopy (XPS) was used to analyze the chemical valence state of elements in Cs_2_SnCl_6_ crystals (Figure 1j). The binding energies of the Cs 3d (Cs 3d_5/2_: 723.55 eV, Cs 3d_3/2_: 737.39 eV), Sn 3d (Sn 3d_3/2_: 495.74 eV, Sn 3d_5/2_: 487.24 eV), and Cl 2p (199.06 eV) peaks are consistent with the reported values [36] (Figure S3), proving that the as-prepared Cs_2_SnCl_6_ crystals are composed of tetravalent Sn. The peaks located at 586.64 eV and 576.38 eV correspond to Te^4+^ 3d, and 170.29 eV to Er^3+^ 4d, respectively. The binding energy of Sn 3d was almost same in un-doped and Te^4+^-doped samples, while it shifted toward a high energy side in the Te^4+^/Er^3+^-co-doped Cs_2_SnCl_6_ (Figure S4). After combining the above ICP-MS results, substitutional site of Sn rather than the interstitial site is likely occupied by Te in the doped sample [37].

### 2.2. Optical Properties

**Figure 2:**
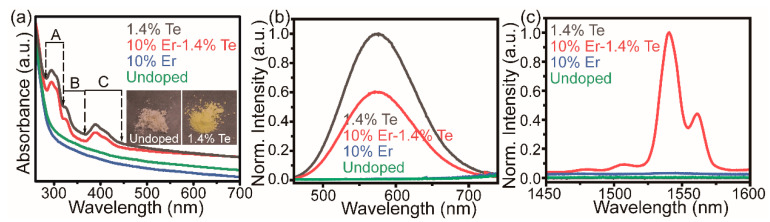
(**a**) UV–Vis absorption spectra of undoped, Er^3+^-doped, Te^4+^-doped, and Te^4+^/Er^3+^-co-doped Cs_2_SnCl_6_ microcrystals. The inset shows photographs of undoped and Te^4+^-doped Cs_2_SnCl_6_ microcrystals. Normalized PL spectra (*λ*_ex_ = 391 nm) of undoped, Er^3+^-doped, Te^4+^-doped and Te^4+^/Er^3+^-co-doped Cs_2_SnCl_6_ microcrystals (**b**) in the visible region and (**c**) in the NIR region.

The optical properties of Te^4+^/Er^3+^-co-doped Cs_2_SnCl_6_ microcrystals were investigated via UV–Vis absorption and photoluminescence (PL) spectra. As shown in Figure 2a, Cs_2_SnCl_6_ microcrystals showed an optical absorption edge at around 315 nm, which is in agreement with the previous report [22]. While Er^3+^-singly doped Cs_2_SnCl_6_ has a similar result to the undoped one, interestingly, Te^4+^-singly doped and Te^4+^/Er^3+^-co-doped Cs_2_SnCl_6_ exhibited intense absorption peaks within the region of 280–450 nm. Compared to the pure white color of the sample without Te^4+^, these new absorption bands changed the hue of the Te^4+^-doped Cs_2_SnCl_6_ to a luminous yellow (see the photographs in the inset of Figure 2a). In accordance with the absorption spectra, the PL excitation (PLE) spectra also showed peaks between 280 and 450 nm (Figure S5). Thereby, the absorption peaks located at 280–320 nm (A), 320–360 nm (B), and 360–450 nm (C) were derived from the Te^4+^-induced ion absorption and could be assigned to the inter-configurational 5s^2^→5s5p transitions of Te^4+^ [36,38].

The PL spectra of undoped and doped Cs_2_SnCl_6_ upon excitation at 391 nm were given in Figure 2b,c. In the visible region, no emission peaks were observed for both the pristine and Er^3+^-doped Cs_2_SnCl_6_ microcrystals (Figure 2b). In contrast, an intense yellow emission at about 577 nm with a large Stokes shift of 127 nm occurs after the doping of Te^4+^ in Cs_2_SnCl_6_, and the luminescence intensity of Te^4+^/Er^3+^-co-doped Cs_2_SnCl_6_ is a little lower than that of the Te^4+^-singly doped one. Meanwhile, no emission was observed in the undoped and Te^4+^-doped Cs_2_SnCl_6_ in the NIR region (Figure 2c). Quite weak NIR emissions of Er^3+^-doped Cs_2_SnCl_6_ were observed in the spectra region from 1450 to 1600 nm, originating from the characteristic ^4^I_13/2_→^4^I_15/2_ transition of the Er^3+^ ion (Figure S6) [38,39]. The NIR emission intensity of Er^3+^-doped Cs_2_SnCl_6_ is too weak to evaluate the PLQY. In sharp contrast, Te^4+^/Er^3+^-co-doped Cs_2_SnCl_6_ displayed an intense NIR emission at 1540 nm and its PLQY was 0.8%. It is also noted that the PL spectrum has other peaks at 1562 nm along with shoulders at around 1480 nm and 1506 nm, which may be caused by the crystal field split manifold of ^4^I_13/2_ and ^4^I_15/2_ states [40]. The phenomenon that the decreased peak intensity at 577 nm accompanies the enhanced emission at 1540 nm in Te^4+^/Er^3+^-co-doped Cs_2_SnCl_6_ as compared with the Te^4+^singly doped sample suggests that the energy transfer and sensitization take place between the luminescent centers Te^4+^ and Er^3+^ in the former.

To achieve the highest NIR emission intensity for Te^4+^/Er^3+^-co-doped Cs_2_SnCl_6_, the Er^3+^ doping concentration was first optimized to 10% via monitoring the NIR emission intensity of Er^3+^-doped Cs_2_SnCl_6_ (Figure S7). Subsequently, the Te^4+^ precursor concentrations were varied while keeping a constant Er^3+^ concentration of 10%. It was seen in Figure S8 that the NIR emission intensity gradually increased to a maximum value at 1.4% Te^4+^ content and then decreased upon increasing the Te^4+^ doping amount (0.2–2.6%). The decrease in PL intensity is due to the concentration quenching effect arising from the energy migration among the ions [39]. As discerned in Figure 2c, Te^4+^-doped Cs_2_SnCl_6_ showed no luminescence at all in the NIR region of 1450–1600 nm. However, the luminescence intensity of Er^3+^ was remarkably enhanced with increasing Te^4+^ concentrations below 1.4%, which confirms the sensitization effect of Te^4+^ on the NIR luminescence of Er^3+^.

**Figure 3:**
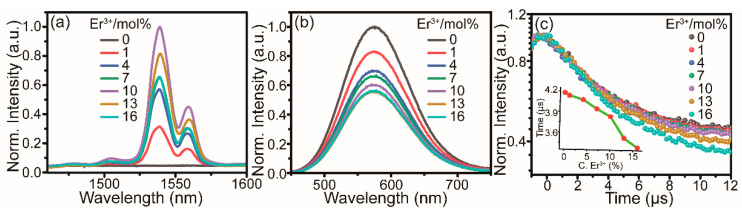
Normalized PL emission spectra (*λ*_ex_ = 391 nm) of 1.4% Te^4+^/x% Er^3+^-co-doped Cs_2_SnCl_6_ microcrystals (**a**) in the NIR region and (**b**) in the visible region. (**c**) Visible luminescence decay curves of 1.4% Te^4+^/x% Er^3+^-co-doped Cs_2_SnCl_6_ (*λ*_ex_ = 391 nm and *λ*_em_ = 577 nm). Inset shows the PL lifetime of 1.4% Te^4+^/x% Er^3+^-co-doped Cs_2_SnCl_6_ for the visible emission as a function of Er^3+^ concentration.

To better understand the sensitization effect of Te^4+^ on the Er^3+^ NIR emission, PL and time-resolved PL (TRPL) measurements of Te^4+^/Er^3+^-co-doped Cs_2_SnCl_6_ were carried out at different Er^3+^ concentrations. As expected, the NIR emission intensity gradually increased with an increasing Er^3+^ concentration from 0 to 10% (Figure 3a), while the visible PL intensity continuously decreased (Figure 3b). The intensity of NIR emissions declines as the Er^3+^ concentration exceeds 10%, which suggests the occurrence of the concentration quenching effect. For Er^3+^-singly doped Cs_2_SnCl_6_, however, different Er^3+^ doping amounts all lead to a negligible NIR luminescence (Figure S9). At the same time, the visible luminescence lifetimes of Te^4+^/Er^3+^-co-doped Cs_2_SnCl_6_ also decreased from 4.18 to 3.39 with the increase in the Er^3+^ concentration (Figure 3c, Table S4), which corresponds to the continuously increasing energy transfer efficiency (1 − τ_x_/τ_0_, where τ_0_ is the lifetime of visible luminescence with Er^3+^ doping amount x = 0 [41].) of Te^4+^/Er^3+^ from 0.96% to 18.9% and benefits intense NIR emissions.

**Figure 4:**
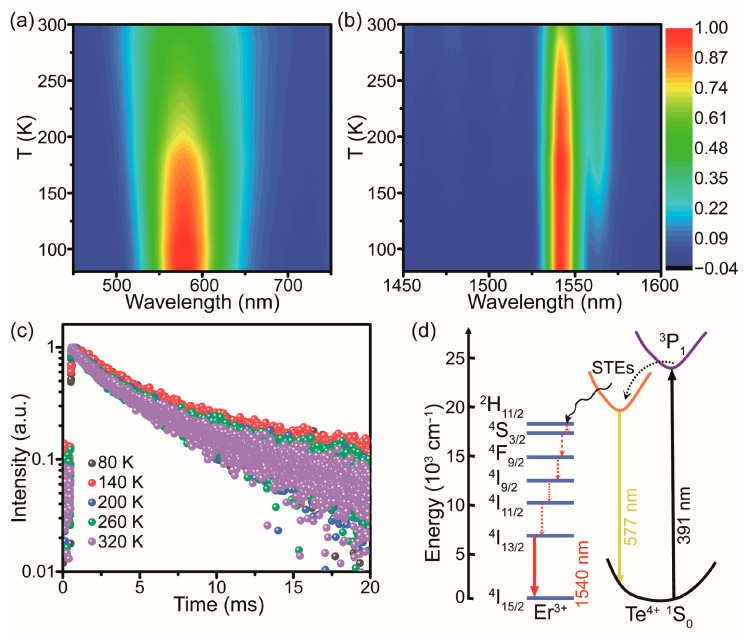
Contour plots of the normalized temperature-dependent PL emission spectra of Te^4+^/Er^3+^-co-doped Cs_2_SnCl_6_ microcrystals under excitation at 391 nm (**a**) in the visible region and (**b**) in the NIR region. (**c**) PL decay curves of 1540 nm emission measured at different temperatures in Te^4+^/Er^3+^-co-doped Cs_2_SnCl_6_ microcrystals (*λ*_ex_ = 391 nm). (**d**) Schematic illustration of luminescence mechanisms in Te^4+^/Er^3+^-co-doped Cs_2_SnCl_6_ microcrystals. The full, dashed, and curve arrows represent the radiative transition, non-radiative transition, and energy transfer process, respectively.

Furthermore, temperature-dependent PL spectra were studied for the as-prepared Te^4+^/Er^3+^-co-doped Cs_2_SnCl_6_. Figure 4a showed that the PL intensities of Te^4+^ declined monotonically upon increasing the temperature from 80 K to 300 K. This is attributed to the increased non-radiative transition probability of Te^4+^ at higher temperatures and the thermal-enhanced energy transfer from Te^4+^ to Er^3+^ [33]. In addition, the full width at half maximum (FWHM) increased as the temperature increased (Figure S10). A high Huang–Rhys factor (S) of 18 was obtained according to the temperature dependence of FWHM, which reveals the strong electron–phonon coupling effect in Cs_2_SnCl_6_:Te and facilitates the formation of STEs [24]. Nevertheless, the integrated PL intensities of Er^3+^ increased slightly with rising temperature (Figure 4b and Figure S11), which was accompanied by small variations in the PL lifetime at 1540 nm within this temperature range (Figure 4c, Table S5). The temperature-stable PL intensity of the NIR emission reflected a good protection of Er^3+^ 4f electrons by the outer electrons in 5s^2^5p^6^ shells [42]. It is worth noting that the intensity ratio of I_1540_/I_1562_ declined with an increasing temperature (Figure S12). This variation is caused by the population redistribution among crystal field split manifolds of ^4^I_13/2_ and ^4^I_15/2_ states at different temperatures, which is consistent with the similar observations made in different Er^3+^-doped hosts [39,43].

According to the above optical results, the energy transfer mechanism was described in Figure 4d based on the energy level alignment of Te^4+^ and Er^3+^. Thanks to the strong electron-phonon coupling in Cs_2_SnCl_6_ with soft lattice, transient elastic lattice deformation occurs upon photogeneration, where excitons tend to be self-trapped due to its lower energy and form self-trapped excitons (STEs) [43]. Therefore, carriers excited from ^1^S_0_ to ^3^P_1_ of Te^4+^ ion under 391 nm excitation relax to form STEs, which then recombine to yield the broad band yellow emission at 577 nm in Te^4+^ doped Cs_2_SnCl_6_. For Er^3+^ singly-doped samples, the electrons in ground state ^4^I_15/2_ transited to excited state ^4^I_13/2_ under low energy excitation, and then generated very weak NIR emission (1540 nm) due to the parity-forbidden transitions within the 4f^N^ configurations [24,40]. In Te^4+^/Er^3+^ co-doped Cs_2_SnCl_6_, however, partial excitation energy was transferred from STEs to the well-matched ^2^H_11/2_ energy level of Er^3+^ ions in addition to the yellow emission [33,37]. The transferred carriers relaxed non-radiatively to the ^4^I_13/2_ energy level, and finally return to the ground states of the ^4^I_15/2_ energy level through radiative transition, resulting in the enhanced 1540 nm NIR emissions at the expense of the weakened yellow luminescence from STEs.

### 2.3. Moisture Stability

Moisture stability is essential for practical applications of perovskite materials. Impressively, the NIR emission was very stable when the samples were exposed to air and even immersed in water. As shown in Figure S13, the XRD pattern of Te^4+^/Er^3+^-co-doped Cs_2_SnCl_6_ microcrystals was basically unchanged after being left in ambient air for 100 days. The PL intensity decreased by only 13% compared to the original data (Figure S14). Moreover, a strong NIR emission of the microcrystals was maintained after being immersed in deionized water for 2 h (Figure S15). Even after the samples were soaked in water for 8 h, the emissions still remained at 30% of the initial level, while the shape and position of XRD peaks remained unchanged (Figure S16). The superior stability in both the structure and NIR luminescence renders Cs_2_SnCl_6_ microcrystals more promising for practical applications relative to the common lead halide perovskites.

## 3. Conclusions

In conclusion, intense and multiple NIR emissions at 1540 nm and 1562 nm were achieved in a Sn-based double perovskite Cs_2_SnCl_6_ through Te^4+^/Er^3+^ co-doping. Under a low energy excitation at 391 nm, the NIR luminescence originating from the 4f→4f transition of Er^3+^ was significantly enhanced due to the effective energy transfer from the ^1^S_0_→^3^P_1_ transition of Te^4+^. Furthermore, the Te^4+^/Er^3+^ Cs_2_SnCl_6_ microcrystals prepared via the simple co-precipitation method exhibited excellent emission and moisture stability. These findings bring novel emissive features to Cs_2_SnCl_6_ double perovskites, thus expanding their optoelectronic properties for future applications, such as NIR biosensors, anti-counterfeit technologies, and optical fiber communication.

## Data Availability

Not applicable.

## Supplementary Materials

The following supporting information can be downloaded at: https://www.mdpi.com/article/10.3390/molecules28083593/s1, Figure S1: Particle size statistics of MCs; Figure S2: Enlarged shifts of (220) peak of MCs; Figure S3: XPS spectra of MCs; Figure S4: Representative XPS spectra of Sn 3d in the MCs; Figure S5: PLE spectra of MCs in the visible region; Figure S6: PL spectra of MCs in the NIR region; Figure S7: PL emission spectra of MCs in the NIR region; Figure S8: PL emission spectra of *x*% Te^4+^-10% Er^3+^ co-doped MCs; Figure S9: NIR intensity variations of MCs with different Er^3+^ concentrations; Figure S10: Fitting results of the FWHM; Figure S11: Integral intensity of NIR peak at different temperatures ; Figure S12: Peak intensity ratio of 1540 nm (I_1540_)/1562 nm (I_1562_) emissions at different temperatures; Figure S13: XRD patterns of MCs before and after their exposure to ambient air; Figure S14: PL emission spectra of MCs before and after their exposure to ambient air; Figure S15: PL emission spectra of MCs before and after their soak in water; Figure S16: XRD patterns of MCs before and after their soak in water; Table S1: Elemental analyses of the MCs by EDS; Table S2: Results of the Te and Er ion ICP-MS test in MCs; Table S3: The calculated lattice parameters of MCs via XRD peaks; Table S4: Average PL lifetimes of MCs with different Er^3+^ concentrations; Table S5: Temperature-dependent PL lifetimes of MCs.

## Sample Availability

Samples of the compounds are not available from the authors.
