# Supplementary material for: Efficient Near-Infrared Luminescence Based on Double Perovskite Cs2SnCl6
Source: Molecules. 2023 Apr 20;28(8):3593. doi: 10.3390/molecules28083593 (PMC10145864; doi:10.3390/molecules28083593)
Supplement: Supplementary file 1 [file molecules-28-03593-s001.zip › molecules-2360845-supplementary.pdf]

Efficient near-infrared luminescence based on double perovskite Cs_2SnCl_6

Xiaofei Qing [1], Chuanli Wu [1,2,*] and Xiuxun Han [1,2,3,*]

[1] Institute of Optoelectronic Materials and Devices, Faculty of Materials Metallurgy and Chemistry, Jiangxi University of Science and Technology, Ganzhou 341000, China
[2] National Rare Earth Function Materials Innovation Center, Ganzhou, 341100， China
[3] Guorui Scientific Innovation Rare Earth Functional Materials (Ganzhou) Co., Ltd., Ganzhou 341000, China
* Correspondence: chuanli_wu@hotmail.com; xxhan@ jxust.edu.cn

Experimental Section

Chemicals and materials: Cs_2CO_3 (99.9%), $SnCl_4$ (99.99%), TeO_2 (99.99%) and $ErCl_3·6H_2O$ (99.9%) were purchased from Aladdin (Shanghai, China). Ethanol and hydrochloric acid (HCl) were analytical grade and purchased from Sinopharm Chemical Reagent Co. (Shanghai, China). All chemicals were used as received without further purification.

Synthesis of Te^{4+} and Er^{3+} doped Cs_2SnCl_6 microcrystals (MCs): Te^{4+} and Er^{3+} singly-doped and Te^{4+}/Er^{3+} co-doped Cs_2SnCl_6 MCs were synthesized via a co-precipitation method. In a typical 1.4% Te^{4+}/10% Er^{3+} MCs (nominal concentrations in mole ratio) synthesis, $SnCl_4$ (0.986 mmol), TeO_2 (0.014 mmol) and $ErCl_3·6H_2O$ (0.1 mmol) were mixed with 15 mL of HCl and 5 mL of ethanol in a 20 mL glass bottle. The mixture was heated to 80 °C with vigorous stirring for 30 min until all the precursors were dissolved. Thereafter, 1 mmol Cs_2CO_3 (dissolved in 2 mL of HCl) was added into the reaction mixture, and the MCs were immediately precipitated. The solution was then kept at 80 °C under vigorous stirring for another 0.5 h to ensure the complete reaction. The products were collected by centrifugation, washed with ethanol three times, and finally dried in an oven at 70 °C for 24 h.

For synthesizing singly-doped Cs_2SnCl_6 MCs with different Te^{4+} concentrations (x% Te^{4+}), x% mmol of TeO_2 and (1-x%) mmol of $SnCl_4$ were used under identical conditions. For synthesizing co-doped Cs_2SnCl_6 MCs with different Er^{3+} concentrations (1.4% Te^{4+}/x% Er^{3+}), 0.986 mmol of $SnCl_4$, 0.014 mmol of TeO_2, and x% mmol of $ErCl_3 \cdot 6H_2O$ were used under identical conditions. For synthesizing Cs_2SnCl_6 MCs with different Te^{4+} concentrations (x% Te^{4+}/10% Er^{3+}), (1-x%) mmol of $SnCl_4$, x% mmol of TeO_2, and 0.1 mmol of $ErCl_3 \cdot 6H_2O$ were used under identical conditions.

Characterization: Powder XRD patterns were collected with an X-ray diffractometer (TD-3700) using Cu Kα1 radiation (λ=0.154187 nm). Inductively coupled plasma-mass spectroscopy (ICP-MS) analyses were conducted on an ICP-MS spectrometer (Agilent 8800). The scanning electron microscopy (SEM) measurements were performed on the Tescan Mira3 equipped with energy-dispersive X-ray spectroscopy (EDS). X-ray photoelectron spectroscopy (XPS) was carried out on a Thermo Fisher Scientific K-Alpha using Al Kα (1486.8 eV). UV-vis absorption spectra were measured on a Shimadzu UV-2600 spectrometer. Photoluminescence (PL) excitation and emission spectra were recorded on the HORIBA FluoroMax spectrometer. PL decay curves were measured on the FLS980 spectrometer (Edinburgh) equipped with both continuous (450 W) and pulsed xenon lamps. The PLQYs were obtained using an FLS1000 spectrometer (Edinburgh). The photographs of the samples were taken using a HongMi K40 cell phone without using any filter.

Table S1. Elemental analyses of the as-synthesized Cs_2SnCl_6: 1.4%Te^{4+}/10%Er^{3+} MCs by EDS.

Sample	Cs : Sn : Te : Er : Cl (molar ratio[%])
Cs_2SnCl_6:1.4% Te^{4+}/10% Er^{3+}	22.73 : 11.76 : 0.13 : 0.24 : 65.14

Table S2 Nominal and actual Te^{4+}/Er^{3+} co-doping concentrations in Cs_2SnCl_6 (1): 1.4% Te^{4+}/10% Er^{3+} MCs. The nominal Er^{3+} doping concentration was defined by the molar ratio of Er to (Sn+Te) in the precursor solution, the nominal Te^{4+} doping concentration was defined by the molar ratio of Te to (Sn+Te) in the precursor solution, and the actual Te^{4+}/Er^{3+} doping concentrations were identified by ICP-MS.

Sample	Nominal		Actual	
	Te^{4+}/mol%	Er^{3+}/mol%	Te^{4+}/mol%	Er^{3+}/mol%
1	1.4	10	1.6	2.0

Table S3 The calculated lattice parameters of undoped, Er^{3+} singly-doped, Te^{4+} singly-doped and Te^{4+}/Er^{3+} co-doped Cs_2SnCl_6 microcrystals via XRD peaks.

Note: a: cell lattice parameters, *V*: cell volume, R_{wp}: weighted profile factor, R_p: the reliability factor of the profile, χ^2: goodness of fit.

Samples	a (Å)	*V* (Å^3)	*R*wp (%)	*R*p (%)	χ^2
Undoped	10.3853	1120.101	10.07	6.83	5.530
10% Er	10.3870	1120.662	10.02	6.86	5.750
1.4% Te	10.3882	1121.050	9.53	6.64	4.680
10% Er-1.4% Te	10.3894	1121.422	10.50	7.02	5.812

Table S4 Average PL lifetimes of Cs_2SnCl_6: 1.4% Te^{4+}/x% Er^{3+} MCs with different Er^{3+} concentrations (x% Er^{3+}) by monitoring the Te^{4+} emissions at 577 nm. All samples show double-exponential decay behavior as the following formula：

$$I_{(t)} = I_0 + A_1 e^{-\frac{t}{\tau_1}} + A_2 e^{-\frac{t}{\tau_2}}$$

The average lifetime τ_{avg} of the fluorescence energy level can be measured by the following formula:

$$\tau_{avg} = \frac{A_1\tau_1^2 + A_2\tau_2^2}{A_1\tau_1 + A_2\tau_2}$$

x mol% Er	τ/μs
0	4.18
1	4.14
4	4.08
7	3.95
10	3.84
13	3.53
16	3.39

Table S5 Temperature-dependent PL decay curves of Cs_2SnCl_6: 1.4%Te^{4+}/10%Er^{3+} MCs were obtained by monitoring the Er^{3+} emission at 1540 nm. The PL lifetime of Er^{3+} remained essentially unchanged (4.0 ms) with the temperature rise

Temperature (K)	Lifetime (ms)
80	4.1
140	4.2
200	4.0
260	3.9
320	3.8

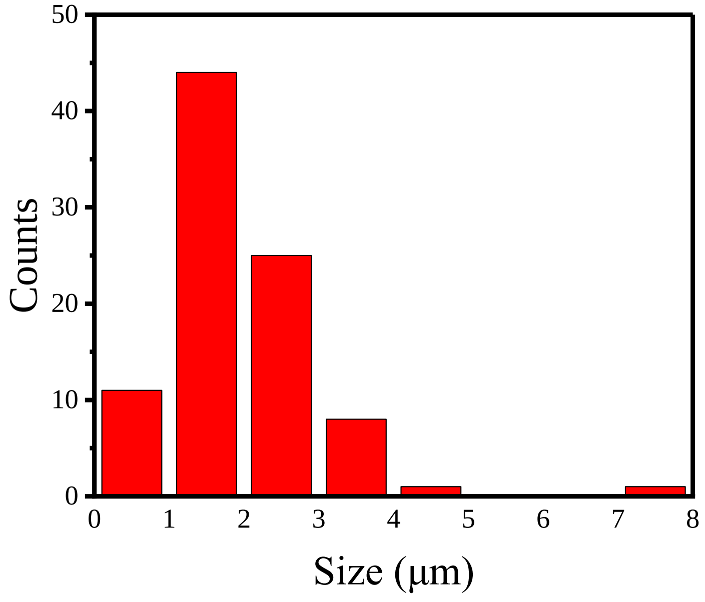

Figure S1. Particle size statistics of Te^{4+}/Er^{3+} co-doped Cs_2SnCl_6 microcrystals.

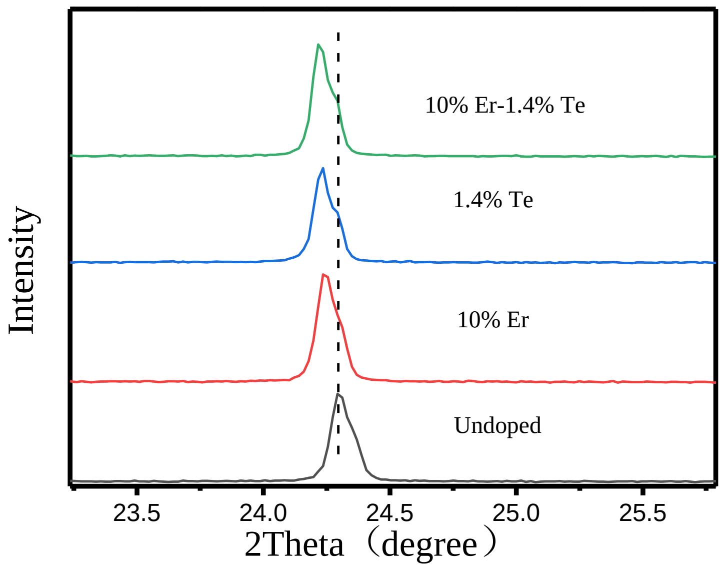

Figure S2. Enlarged shifts of (220) peak of the undoped, Te^{4+} singly-doped, Er^{3+} singly-doped and Te^{4+}/Er^{3+} co-doped Cs_2SnCl_6 MCs.

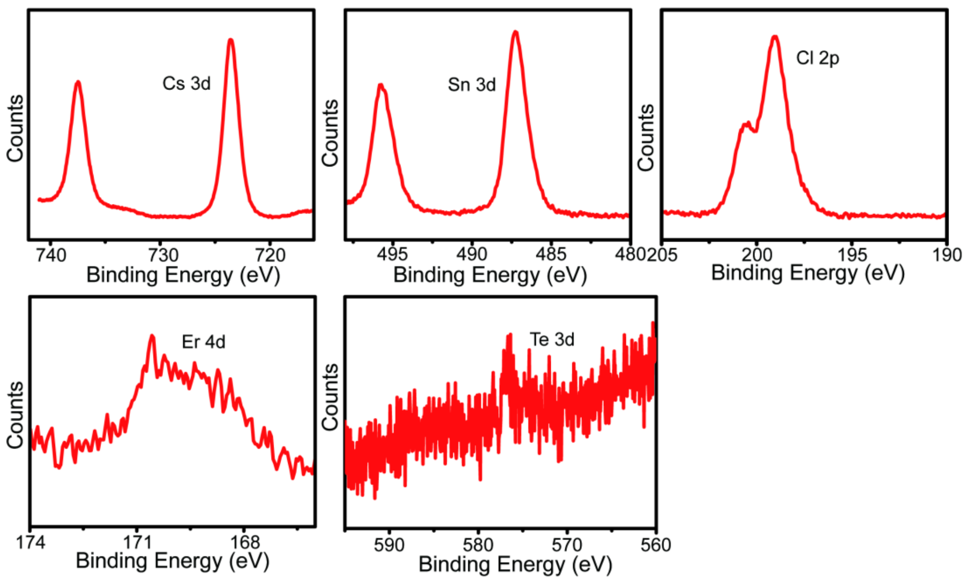

Figure S3. XPS spectra of Te^{4+}/Er^{3+} co-doped Cs_2SnCl_6 microcrystals.

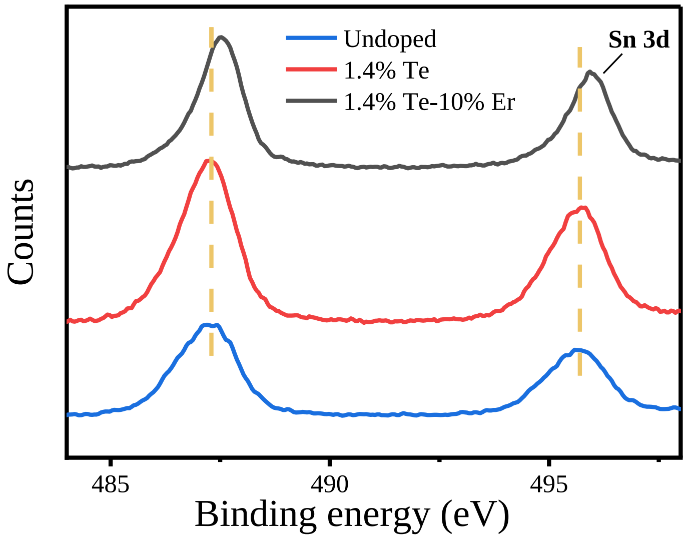

Figure S4. Representative XPS spectra of Sn 3d in the undoped, Te^{4+} singly-doped and Te^{4+}/Er^{3+} co-doped Cs_2SnCl_6 MCs.

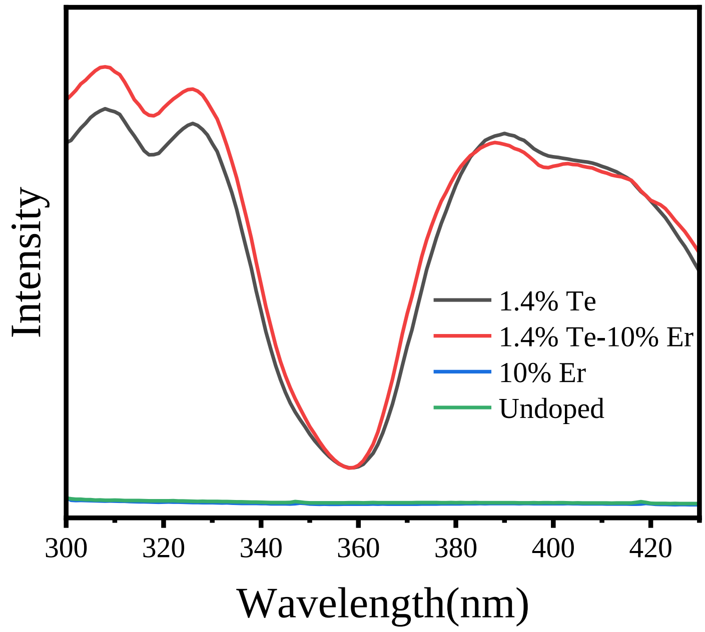

Figure S5. PLE spectra (λ_{em}=577 nm) of undoped, Er^{3+} doped, Te^{4+} doped and Te^{4+}/Er^{3+} co-doped Cs_2SnCl_6 microcrystals in the visible region.

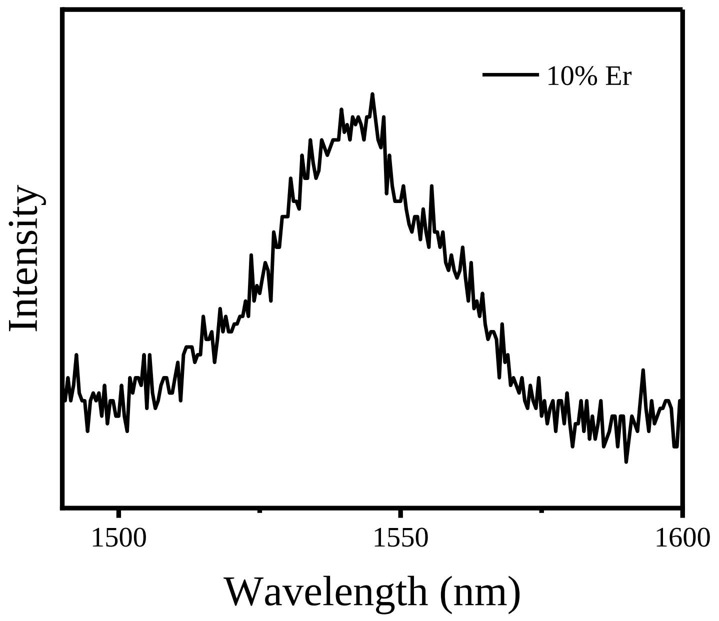

Figure S6. PL spectra (λ_{ex}=391 nm) of 10% Er^{3+} doped Cs_2SnCl_6 microcrystals in the NIR region.

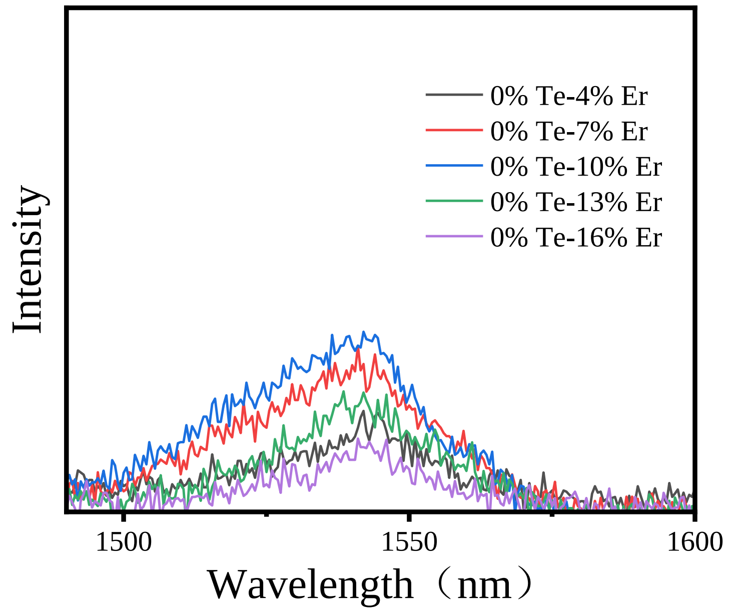

Figure S7. PL emission spectra (λ_{ex}=391 nm) of *x*% Er^{3+} doped Cs_2SnCl_6 microcrystals in the NIR region.

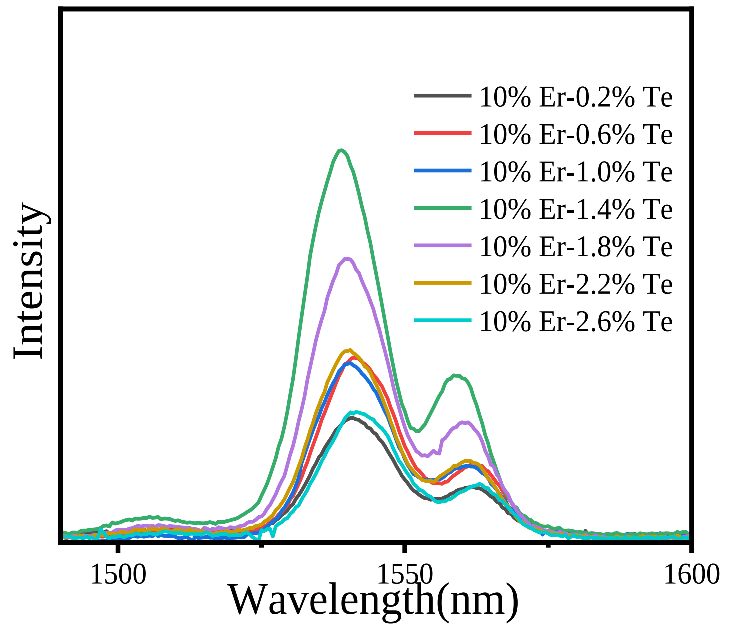

Figure S8. PL emission spectra (λ_{ex}=391 nm) of *x*% Te^{4+}-10% Er^{3+} co-doped Cs_2SnCl_6 microcrystals.

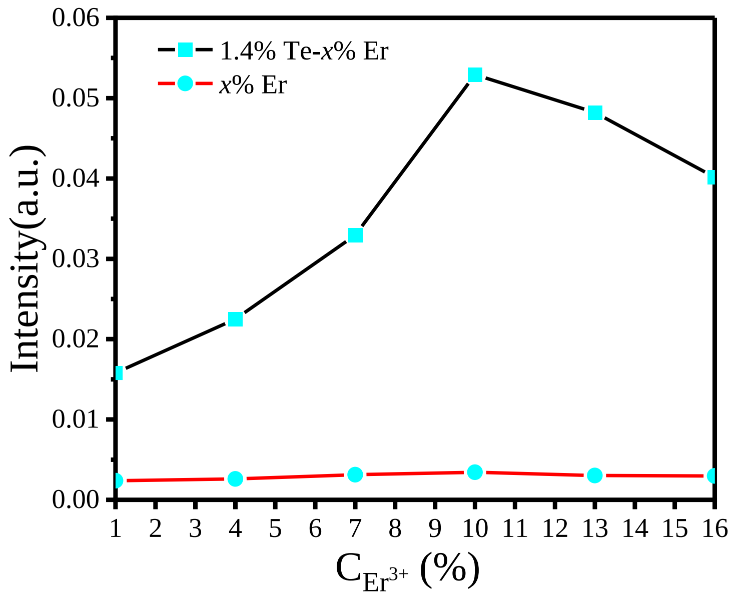

Figure S9. NIR intensity variations of Er^{3+} singly doped and 1.4%Te^{4+}-Er^{3+}co-doped samples with different Er^{3+} concentrations ($C_{Er}{}^{3+}$ (%)).

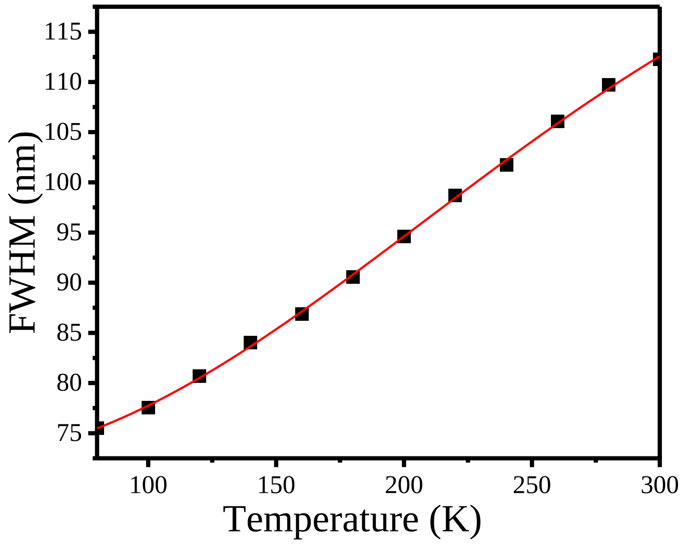

Figure S10 Fitting results of the FWHM as a function of temperature.

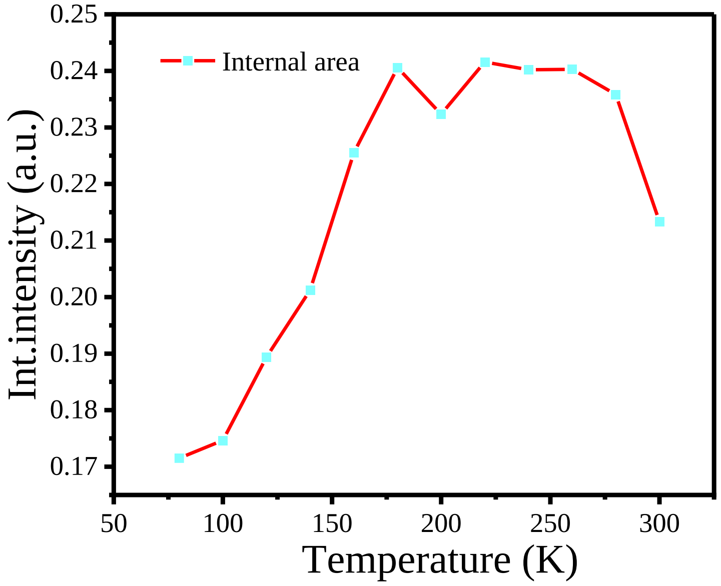

Figure S11. Integral intensity of NIR peak at different temperatures (80-300 K).

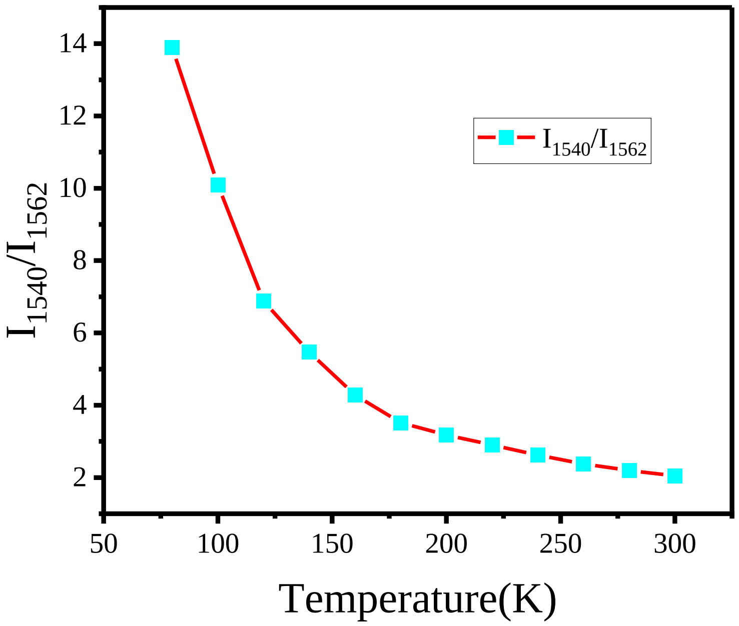

Figure S12. Peak intensity ratio of 1540 nm (I_{1540})/1562 nm (I_{1562}) emissions at different temperatures.

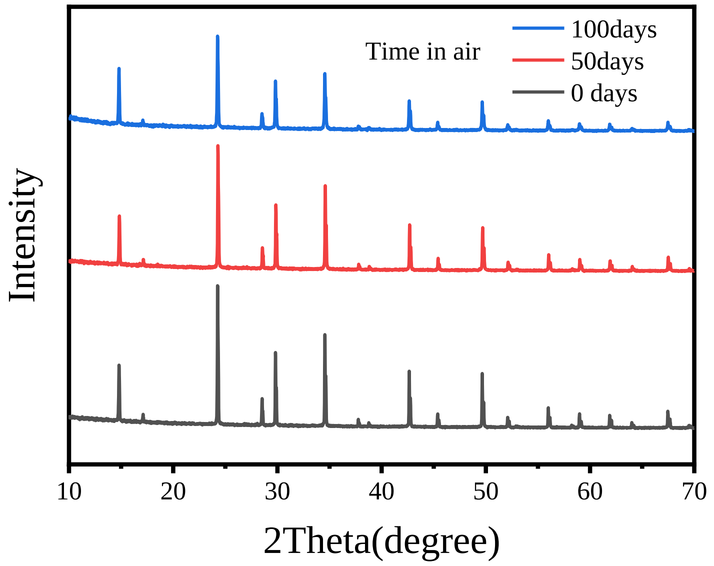

Figure S13. XRD patterns of Cs_2SnCl_6:1.4% Te^{4+}/10% Er^{3+} MCs before and after their exposure to ambient air for 50 and 100 days. The XRD patterns remain essentially unchanged, manifesting the excellent air stability of the MCs.

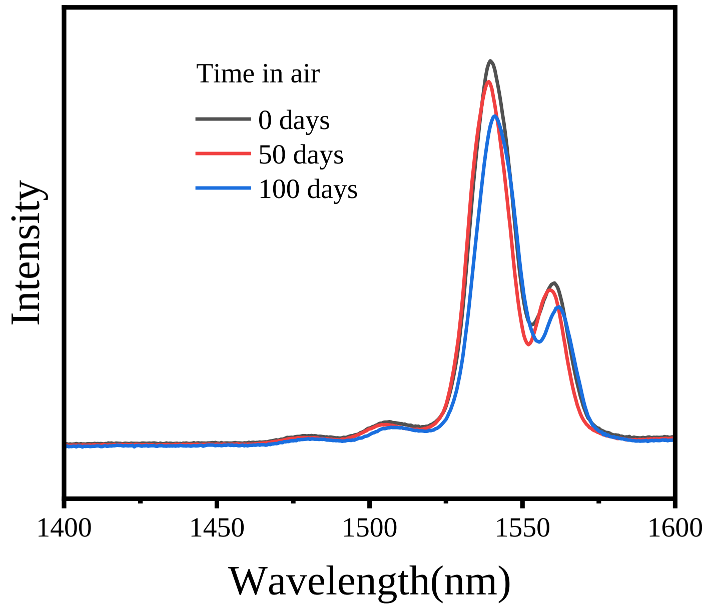

Figure S14. PL emission spectra (λ_{ex}=391 nm) of Cs_2SnCl_6:1.4% Te^{4+}/10% Er^{3+}MCs before and after their exposure to ambient air for 50 and 100 days. The spectra curves of the MCs remained essentially unchanged after their storage in ambient air for 100 days, except for a slight decrease in the PL intensities, manifesting the striking air stability of the MCs.

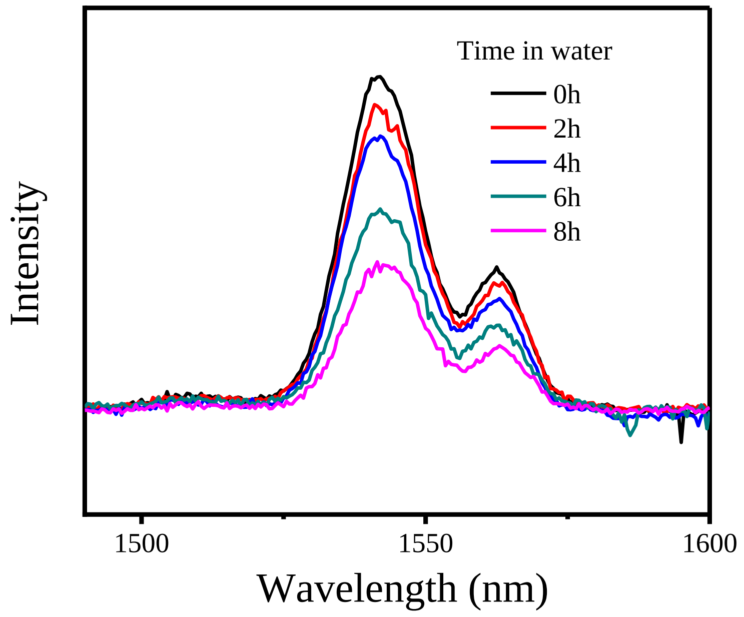

Figure S15. PL emission spectra (λ_{ex}=391 nm) of Cs_2SnCl_6: 1.4% Te^{4+}/10% Er^{3+} MCs before and after their soak in water for 2-8 h.

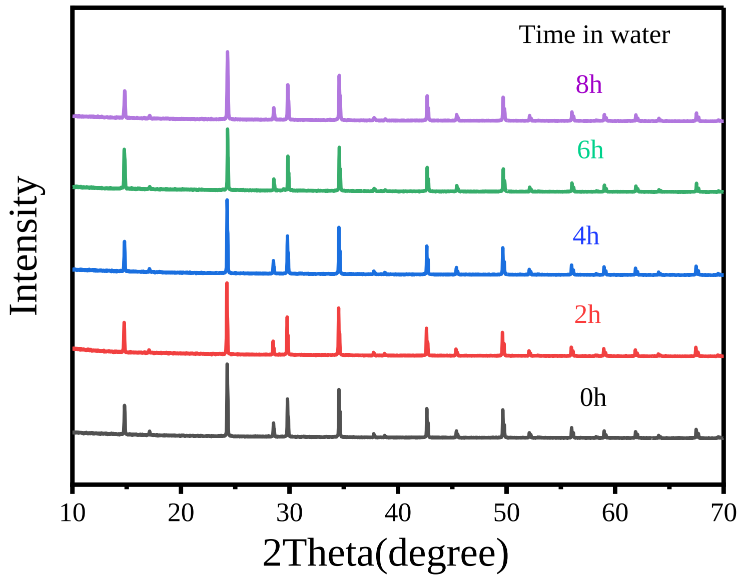

Figure S16. XRD patterns of Cs_2SnCl_6: 1.4% Te^{4+}/10% Er^{3+} MCs before and after their soak in water for 2-8 h.
